# Photoconversion and Fluorescence Properties of a Red/Green-Type Cyanobacteriochrome AM1_C0023g2 That Binds Not Only Phycocyanobilin But Also Biliverdin

**DOI:** 10.3389/fmicb.2016.00588

**Published:** 2016-04-26

**Authors:** Keiji Fushimi, Takahiro Nakajima, Yuki Aono, Tatsuro Yamamoto, Ni-Ni-Win, Masahiko Ikeuchi, Moritoshi Sato, Rei Narikawa

**Affiliations:** ^1^Department of Biological Science, Faculty of Science, Shizuoka UniversityShizuoka, Japan; ^2^Graduate School of Arts and Sciences, University of TokyoTokyo, Japan; ^3^Core Research for Evolutional Science and Technology, Japan Science and Technology AgencySaitama, Japan

**Keywords:** optogenetics, linear tetrapyrrole, GFP, near-infrared fluorescence, live cell imaging

## Abstract

Cyanobacteriochromes (CBCRs) are distantly related to the red/far-red responsive phytochromes. Red/green-type CBCRs are widely distributed among various cyanobacteria. The red/green-type CBCRs covalently bind phycocyanobilin (PCB) and show red/green reversible photoconversion. Recent studies revealed that some red/green-type CBCRs from chlorophyll *d*-bearing cyanobacterium *Acaryochloris marina* covalently bind not only PCB but also biliverdin (BV). The BV-binding CBCRs show far-red/orange reversible photoconversion. Here, we identified another CBCR (AM1_C0023g2) from *A. marina* that also covalently binds not only PCB but also BV with high binding efficiencies, although BV chromophore is unstable in the presence of urea. Replacement of Ser334 with Gly resulted in significant improvement in the yield of the BV-binding holoprotein, thereby ensuring that the mutant protein is a fine platform for future development of optogenetic switches. We also succeeded in detecting near-infrared fluorescence from mammalian cells harboring PCB-binding AM1_C0023g2 whose fluorescence quantum yield is 3.0%. Here the PCB-binding holoprotein is shown as a platform for future development of fluorescent probes.

## Introduction

Phytochromes and cyanobacteriochromes (CBCRs) are photoreceptors that form a large superfamily with a linear tetrapyrrole-binding GAF (cGMP-phosphodiesterase/adenylate cyclase/FhlA) domain (Ikeuchi and Ishizuka, 2008; Anders and Essen, 2015). Some CBCRs are known to be involved in regulation of light acclimation processes such as phototaxis (Yoshihara et al., 2000; Narikawa et al., 2011; Song et al., 2011; Savakis et al., 2012; Campbell et al., 2015), chromatic acclimation (Kehoe and Grossman, 1996; Hirose et al., 2010) and light-dependent cell aggregation (Enomoto et al., 2014, 2015). Only GAF domain of CBCRs is enough for chromophore ligation and photoconversion, although additional PAS (Per/Arnt/Sim) and PHY (phytochrome-specific) domains in phytochromes are necessary. CBCRs have been roughly categorized into two types according to the chromophore they contain in the thermostable state: Phycoviolobilin (PVB) and PCB. In both cases, PCB is initially incorporated into the GAF domain and Cys residue within the GAF domain covalently ligates to C3^1^ of the chromophore. PVB-binding CBCRs sense relatively shorter wavelength light covering ultraviolet-to-green region (Yoshihara et al., 2004; Ishizuka et al., 2006; Rockwell et al., 2008, 2012a,b; Narikawa et al., 2011; Song et al., 2011; Enomoto et al., 2012; Ma et al., 2012; Cho et al., 2015), whereas PCB-binding CBCRs sense longer wavelength light covering ultraviolet-to-red region (Hirose et al., 2008, 2013; Narikawa et al., 2008a,b, 2014; Rockwell et al., 2011, 2012c; Chen et al., 2012). These CBCRs commonly show light-induced *Z*/*E* isomerization of a double bond between rings C and D, followed by various structural changes such as reversible protochromic cycle and reversible Cys-adduct formation (Rockwell et al., 2008; Burgie et al., 2013; Hirose et al., 2013; Narikawa et al., 2013, 2014).

Among the PCB-binding CBCRs, red/green-type CBCRs are widely spread among various cyanobacteria and most extensively analyzed so far (Narikawa et al., 2008a, 2013; Fukushima et al., 2011; Chen et al., 2012; Kim et al., 2012a,b,c; Rockwell et al., 2012c, 2015a,b; Velazquez Escobar et al., 2013; Slavov et al., 2015; Song et al., 2015a,b). The red/green-type CBCRs show reversible photoconversion between a red-absorbing form (Pr) with 15*Z*-PCB and a green-absorbing form (Pg) with 15*E*-PCB. Structure of Pr form provides direct insights into chromophore–protein interaction (Narikawa et al., 2013). Detailed spectroscopic analyses based on this structure have revealed their photoconversion mechanism in which excited state destabilization and ring D distortion are suggested to occur to form blue-shifted Pg form upon red-light irradiation (Rockwell et al., 2014; Song et al., 2015a).

Recently, it has been revealed that red/green-type CBCRs (AM1_1557g2 and AM1_1870g3) derived from the chlorophyll *d*-bearing cyanobacterium *Acaryochloris marina* covalently bind not only PCB but also biliverdin (BV; Narikawa et al., 2015a,b). BV-binding ones show reversible photoconversion between far red-absorbing (Pfr) form and orange-absorbing (Po) form, whereas PCB-binding ones show normal red/green reversible photoconversion. Site-directed mutagenesis suggests that a Cys residue within the GAF domain covalently ligates not only to PCB but also to BV. BV is present in most organisms including mammals and far-red light can penetrate into deep tissues, with a potential as optogenetic and bioimaging tools (Ziegler and Möglich, 2015).

Here, we report another GAF domain (second GAF domain of AM1_C0023 called AM1_C0023g2) from *A. marina* that covalently binds not only PCB but also BV. Replacement of Ser334 with Gly resulted in significant improvement in yield of the BV-binding holoprotein. Further, we detected near-infrared fluorescence from mammalian cells harboring AM1_C0023g2-PCB whose fluorescence quantum yield is 3.0%.

## Materials and Methods

### Plasmid Construction

The nucleotide sequence of AM1_C0023g2 was cloned into pET28a (Novagen) using In-fusion HD Cloning kit (TaKaRa) as described previously (Narikawa et al., 2015b). AM1_C0023g2 sequence was amplified by polymerase chain reaction (PCR) with a specific primer set (5′-CGCGGCAGCCATATGAATATTTCCGAGATTATT-3′, 5′-CTCGAATTCGGATCCTCAAGCTTCTGCTTTGTTTTT-3′) and PrimeSTAR Max DNA polymerase (TaKaRa). The inserted sequence was confirmed by sequencing with an ABI310 genetic analyzer. Replacement of AM1_C0023g2 Ser334 with Gly (denoted S334G) was performed using a specific primer set (5′-CAACAAGGATATACAGATTGTCATCTA-3′, TGTATATCCTTGTTGATAAATGTCAGC) and PrimeSTAR max DNA polymerase as described previously.

To construct GFP-fused AM1_C0023g2 and AM1_1557g2, the nucleotide sequences of GFP, AM1_C0023g2 and AM1_1557g2 were amplified by PCR with specific primer sets and Pyrobest DNA polymerase (TaKaRa). The following primer sets were used to introduce restriction enzyme sites, a flexible peptide linker sequence, and Kozak sequence: for GFP, 5′-ATGCAAGCTTGCCACCATGGTGAGCAAGGGCGAG-3′ and 5′-GCATCTCGAGACCTCCGCTACCGCCCTTGTACAGCTCGTC-3′; for AM1_C0023g2 and AM1_1557g2, 5′-ATGCCTCGAGAGCGGCCTGGTGCCGCGC-3′ and 5′-GAGCTCGAATTCGGATCC-3′. All sequences were confirmed by sequencing with an ABI 310 genetic analyzer. These constructs were cloned into a mammalian expression vector pcDNA 3.1 (+) (Invitrogen) using the restriction enzyme sites. All the mammalian expression plasmids were purified using QIAGEN plasmid kit (Qiagen).

### Expression, Purification, and SDS-PAGE

His-tagged AM1_C0023g2 was expressed in both BV- and PCB-producing *Escherichia coli* (C41 harboring pKT270 and pKT271, respectively). The His-tagged proteins were isolated by Ni-affinity chromatography as described previously (Narikawa et al., 2015b). The purified proteins were subjected to SDS-PAGE, followed by in-gel fluorescent assay and Coomassie Brilliant Blue staining as described previously (Narikawa et al., 2015b). Fluorescence was visualized through a 600 nm long path filter upon excitation with wavelength of blue (aaa_max_ = 470 nm) and green light (aaa_max_ = 530 nm) through a 562 nm short path filter (WSE-6100 LuminoGraph, WSE-5500 VariRays; ATTO).

### Estimation of Binding Efficiencies

Protein concentration was determined by standard Bradford method (Bio-Rad). We determined extinction coefficients of free PCB (Santa Cruz Biotechnology Inc.) and free BV (Frontier Scientific) under both acidic urea and 1% SDS conditions. Extinction coefficients of the free PCB and BV under the acidic urea condition were calculated to be a little less than 30000, that is comparable to that reported previously (Glazer and Fang, 1973). On the other hand, extinction coefficients of the free PCB and BV under the 1% SDS condition were calculated to be around 17000. Although extinction coefficients of chromophores under the 1% SDS condition tend to be lower than those under the acid urea condition, PCB and BV behave similarly under both conditions. Thus, concentration of PCB bound to proteins was determined by absorbance at 666 nm when denatured with 8 M urea (pH2.0). Similarly that of BV bound to proteins was determined by absorbance at 646 nm when denatured with 1% SDS.

### *In Vitro* Reconstitution

S334G protein that is a mutant of AM1_C0023g2 was used for *in vitro* reconstitution analysis because of its high yield of *in vivo* reconstitution system (see **Figure 4**). Free BV and the apo-S334G protein were mixed in roughly equimolar amounts and incubated for 30 min at 37°C. After removal of unbound BV by spin column, the sample was subjected to spectral analyses.

### Spectroscopy

Ultraviolet and visible absorption spectra were recorded with a Shimadzu UV-2600 spectrophotometer. Monochromic light of various wavelengths for photoconversion was generated using a variable wavelength light source (Opto-Spectrum Generator, Hamamatsu Photonics, Inc.). Acid denaturation of the proteins was performed with 8 M urea, pH 2.0 under the dark condition. Fluorescence spectra were recorded with a StellarNet SILVER-Nova spectrometer (StellerNet, Inc.). Fluorescence quantum yields were measured with Quantaurus-QY (Hamamatsu Photonics, Inc.).

### Imaging of GFP-fused AM1_C0023g2 and AM1_1557g2 in Mammalian Cells

Human cervical carcinoma (HeLa) cells (ATCC, CCL-2) were cultured in Eagle’s minimum essential medium (Sigma) supplemented with 2 mM L-glutamine (Gibco), 10% fetal bovine serum (Gibco), 100 U/mL penicillin and 100 μg/mL streptomycin (Gibco) at 37°C in 5% (v/v) CO_2_. The day before transfection, HeLa cells were plated onto 35-mm glass-based dishes (Iwaki). Transfection was performed using lipofectamine 3000 (Life Technologies) according to the manufacturer’s instructions. Forty-eight hours after transfection, the cells were washed with Hank’s balanced salt solution (HBSS; Gibco) and replaced with HBSS containing either 20 μM PCB (Frontier Scientific) diluted from a stock solution of 20 mM in dimethyl sulfoxide (DMSO; Wako) or 0.1% (v/v) DMSO as the vehicle control. After incubation for 4 h, the cells were washed and replaced with HBSS, and imaged using a Zeiss LSM 710 confocal microscope equipped with a Plan-Apochromat 63×/1.4 oil immersion objective (Carl Zeiss), a 488 nm Ar laser, and a 633 nm He/Ne laser. Green fluorescence of GFP was detected at 493–538 nm upon excitation with the 488 nm laser (6.5 μW measured at the back aperture of the objective lens). Near-infrared fluorescence of CBCR was detected at 638–740 nm upon excitation with the 633 nm laser (67 μW measured at the back aperture of the objective lens). Data analysis was performed using ZEN 2009 (Carl Zeiss).

## Results and Discussion

### AM1_C0023g2 Isolated from PCB-Producing and BV-Producing *E. coli*

**FIGURE 1:**
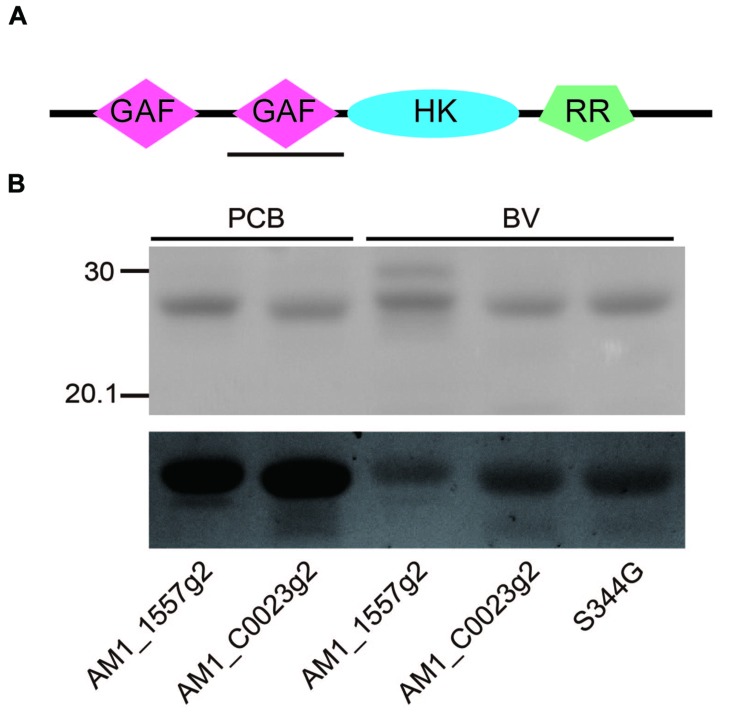
**(A)** Domain structure of the full-length gene product, AM1_C0023. GAF: cGMP-phosphodiesterase/adenylate cyclase/FhlA domain, HK, Histidine kinase domain; RR, response regulator domain. Expressed second GAF domain, AM1_C0023g2, was highlighted by black solid underline. **(B)** SDS-PAGE analyses of PCB- and BV-binding AM1_1557g2 and AM1_C0023g2. Upper gel image: Coomassie Brilliant Blue stained gel. Lower gel image: Linear tetrapyrroles covalently bound to CBCR GAF domains were detected by in-gel fluorescence imaging. The Zn^2+^-staining was performed as previously described (Berkelman and Lagarias, 1986). The gel was directly subjected to fluorescence detection. The gel image was provided with black-and-white inversion for easy detection of fluorescent bands.

We found that AM1_C0023 is closely related in-paralog of AM1_1557. AM1_C0023 is 71% identical to and has a domain architecture same as AM1_1557 (**Figure 1A**). Second GAF domain of AM1_C0023 (AM1_C0023g2) is 84% identical to that of AM1_1557 (AM1_1557g2). Residues highly conserved among red/green-type CBCRs and important for chromophore ligation are also conserved in AM1_C0023g2.

**FIGURE 2:**
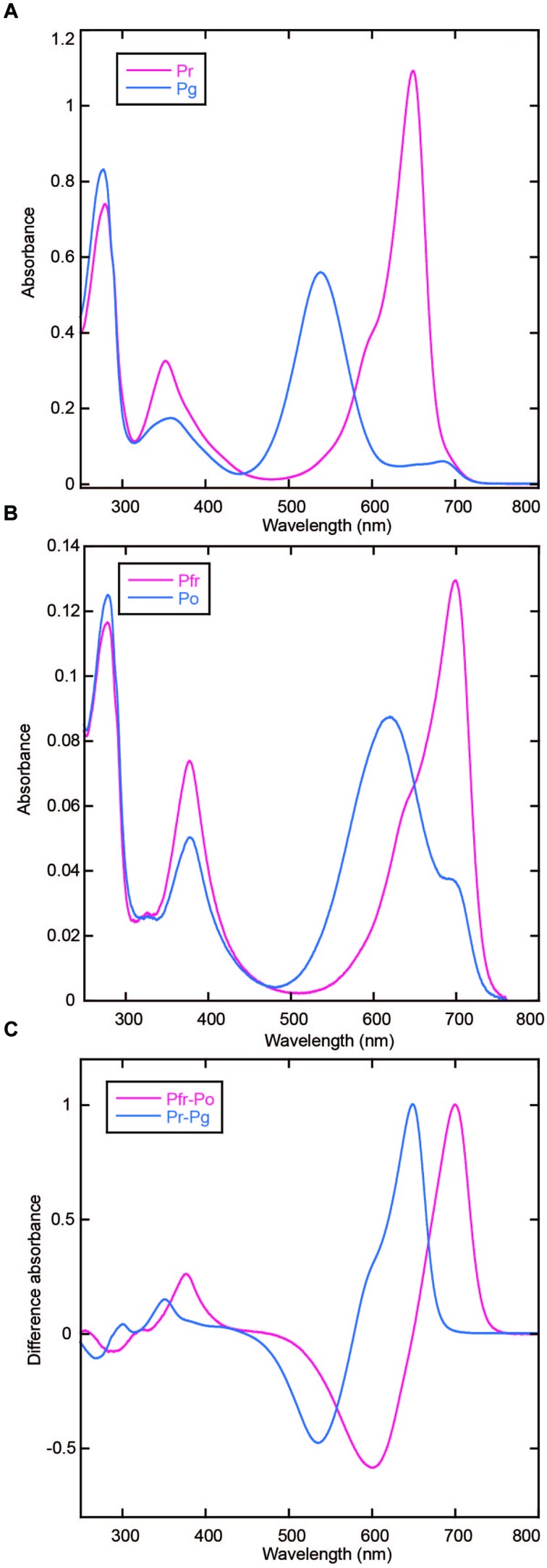
**Photoconversion of AM1_C0023g2-PCB and AM1_C0023g2-BV. (A)** Absorption spectra of Pr (magenta) and Pg (blue) forms of AM1_C0023g2-PCB. **(B)** Absorption spectra of Pfr (magenta) and Po (blue) forms of AM1_C0023g2-BV. **(C)** Difference spectra of PCB- (blue) and BV-binding (magenta) AM1_C0023g2 before and after photoconversion.

AM1_C0023g2 was expressed in both PCB- and BV-producing *E. coli* and purified by using nickel-affinity column chromatography. In-gel fluorescence analysis revealed that AM1_C0023g2 covalently binds not only PCB but also BV like AM1_1557g2 (**Figure 1B**). PCB-binding AM1_C0023g2 (AM1_C0023g2-PCB) showed reversible photoconversion between red-absorbing Pr form at 650 nm and green-absorbing Pg form at 539 nm (**Figure 2A**), whereas BV-binding AM1_C0023g2 (AM1_C0023g2-BV) showed reversible photoconversion between far red-absorbing Pfr form at 699 nm and orange-absorbing Po form at 618 nm (**Figure 2B**). These spectral features are almost same as those of AM1_1557g2 (Narikawa et al., 2015b). Pfr-minus-Po difference spectrum of AM1_C0023g2-BV possesses positive peaks at 700 and 377 nm and negative peaks at 601 and 284 nm, whereas Pr-minus-Pg difference spectrum of AM1_C0023g2-PCB possesses positive peaks at 650, 352, and 301 nm and negative peaks at 536 and 270 nm (**Figure 2C**).

**FIGURE 3:**
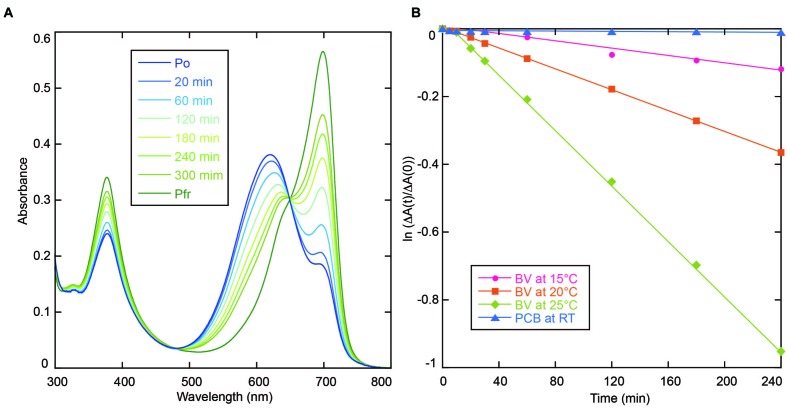
**(A)** Absorption spectra of AM1_C0023g2-BV recorded during dark reversion at 25°C. **(B)** Dark reversion kinetics of PCB- (blue triangle for room temperature) and BV-binding (magenta circle for 15°C, orange square for 20°C and green diamond for 25°C) AM1_C0023g2 from metastable Pg and Po forms to thermostable Pr and Pfr forms, respectively.

AM1_C0023-BV Pfr showed dark reversion to Po (**Figure 3A**), whereas dark reversion of AM1_C0023-PCB Pr was hardly detected (**Figure 3B**). Half-lives for the AM1_C0023-BV during dark reversion measured at 15, 20, and 25°C were 1356, 478, and 180 min, respectively (**Figure 3B**). Slower dark reversion was observed under lower temperature. Hardly detectable and relatively fast dark reversions of PCB- and BV-binding ones, respectively, are also consistent with those found for AM1_1557g2 and AM1_1870g3 (Narikawa et al., 2015a,b).

### Site-Directed Mutagenesis of AM1_C0023g2

**FIGURE 4:**
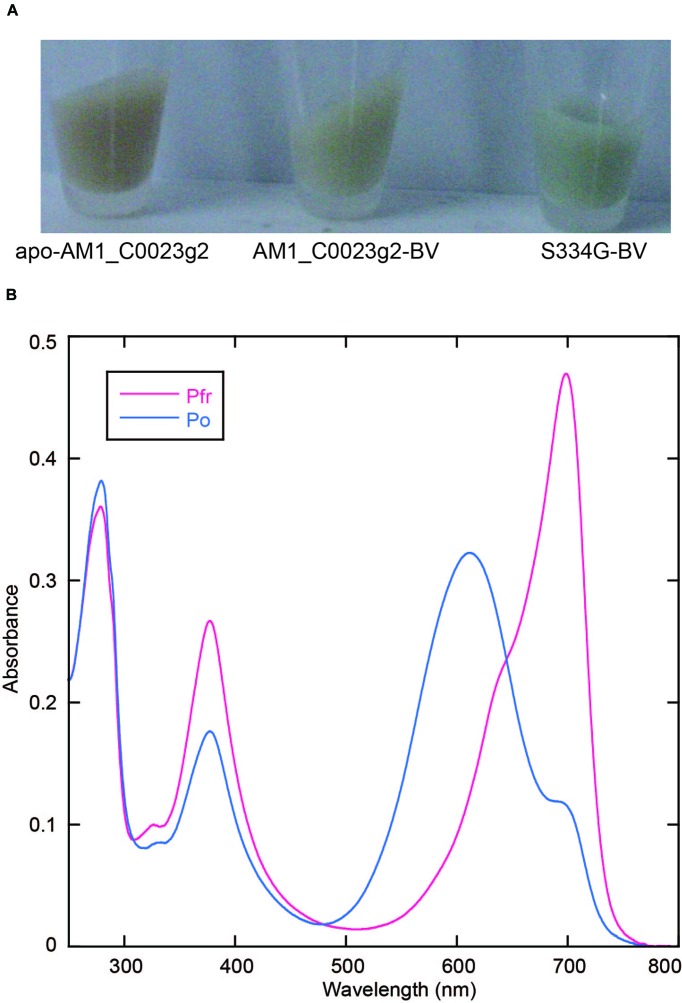
**Replacement of Ser334 of AM1_C0023g2 with Gly. (A)** Photograph of cell pellets harboring apo-AM1_C0023g2, AM1_C0023g2-BV, and S334G-BV. **(B)** Absorption spectra of the Pfr and Po forms of S334G-BV.

In the process of site-directed mutagenesis analysis to obtain proteins with higher fluorescence quantum yields, we incidentally obtained a unique mutant protein, S334G in which Ser334 was replaced with Gly. This replacement resulted in significant improvement in expression yield of BV-binding holoproteins in *E. coli* (**Figure 4A**). Colonies were picked up and grown in small-scale culture. Cell pellet of *E. coli* expressing only the wild type protein showed brown color typical of normal *E. coli*. Cell pellet of *E. coli* expressing both the wild type protein and the heme oxygenase showed pale green color. On the other hand, cell pellet of *E. coli* expressing both the S334G protein and the heme oxygenase showed deep green color. The BV-binding S334G (S334G-BV) showed reversible photoconversion between Pfr at 699 nm and Po at 611 nm (**Figure 4B**). Its Po form is about 7 nm blue-shifted than that of the wild type. Binding efficiency to BV was calculated to be 1.25-fold higher than that of the wild type protein (see Evaluation of Chromophore Binding Efficiencies). In total, judging from the Pfr absorption peak, recovery of the S334G-BV was about five-fold higher than that of the BV-binding wild type from the same culture volume. Although Gly317 of AnPixJg2 corresponding to Ser334 of AM1_C0023g2 does not directly interact with PCB, Gly317 is placed 4 residues upstream of Cys321 that covalently ligates to PCB (Narikawa et al., 2013). This replacement may indirectly affect chromophore-binding pocket to facilitate protein expression and chromophore incorporation. Apart from the underlying mechanism, we consider that S334G-BV is a good platform for further development of optogenetic switches. Slow dark recovery kinetics and almost full photoconversion (90% estimated from the absorption spectra) are favorable for long-term and acute regulation.

### Chromophore Species and Configuration

**FIGURE 5:**
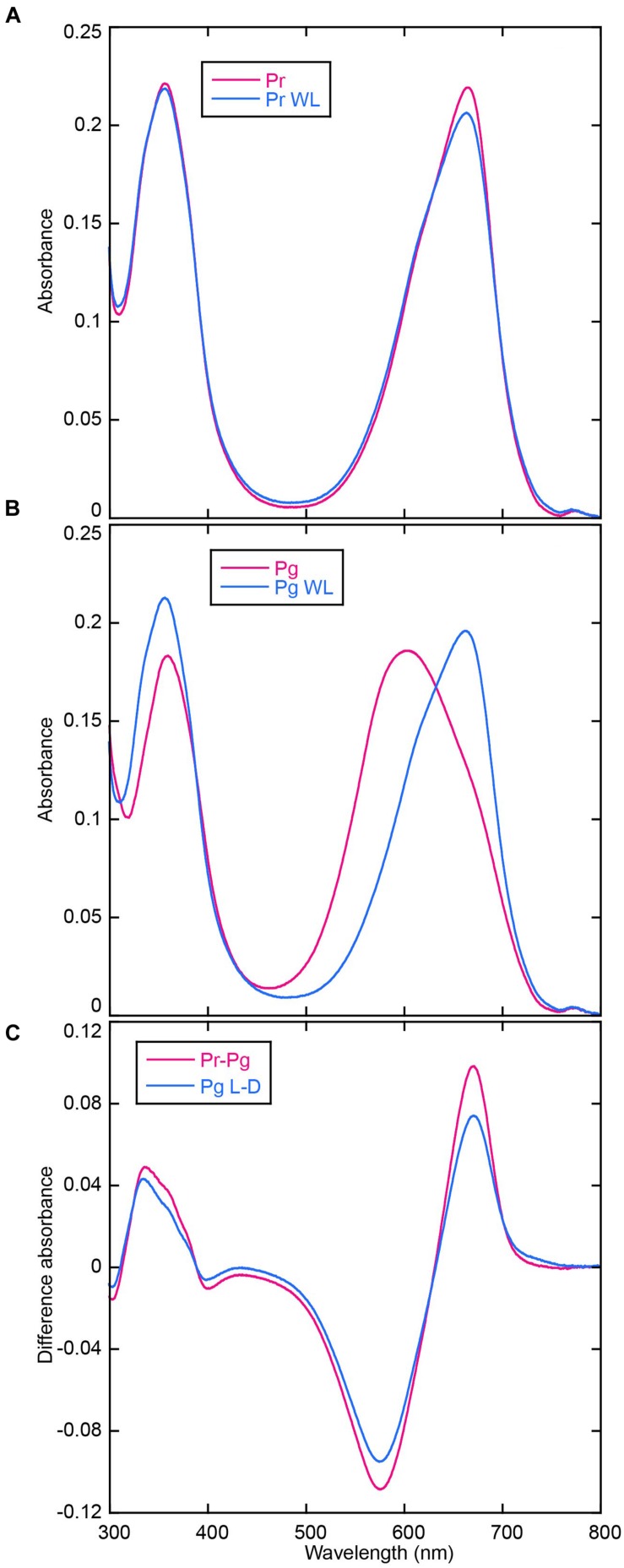
**Acid denaturation of AM1_C0023g2-PCB. (A)** Absorption spectra of acid-denatured AM1_C0023g2-PCB Pr. Absorption spectra just after denaturation (Pr, magenta) and after white light illumination (Pr WL, blue). **(B)** Absorption spectra of acid-denatured AM1_C0023g2-PCB Pg. Absorption spectra just after denaturation (Pg, magenta) and after white light illumination (Pg WL, blue). **(C)** Pr-minus-Pg difference spectrum (Pr-Pg, magenta) and that during photoconversion of denatured Pg (Pg L-D, blue).

Acid-denatured spectra proved that AM1_C0023g2 isolated from PCB-producing *E. coli* undoubtedly bound PCB (**Figure 5**). Absorption maximum of denatured Pr and Pg were observed at 665 and 603 nm, respectively (**Figures 5A,B**). Irradiation of denatured Pg with white light resulted in red shift of the absorbance that is almost identical to that of denatured Pr, whereas irradiation of denatured Pr with white light resulted in no significant change (**Figures 5A,B**). Pr-minus-Pg difference spectrum fits to that during photoconversion of denatured Pg (**Figure 5C**). These results clearly show that Pr and Pg bound 15*Z*- and 15*E*-PCB, respectively.

**FIGURE 6:**
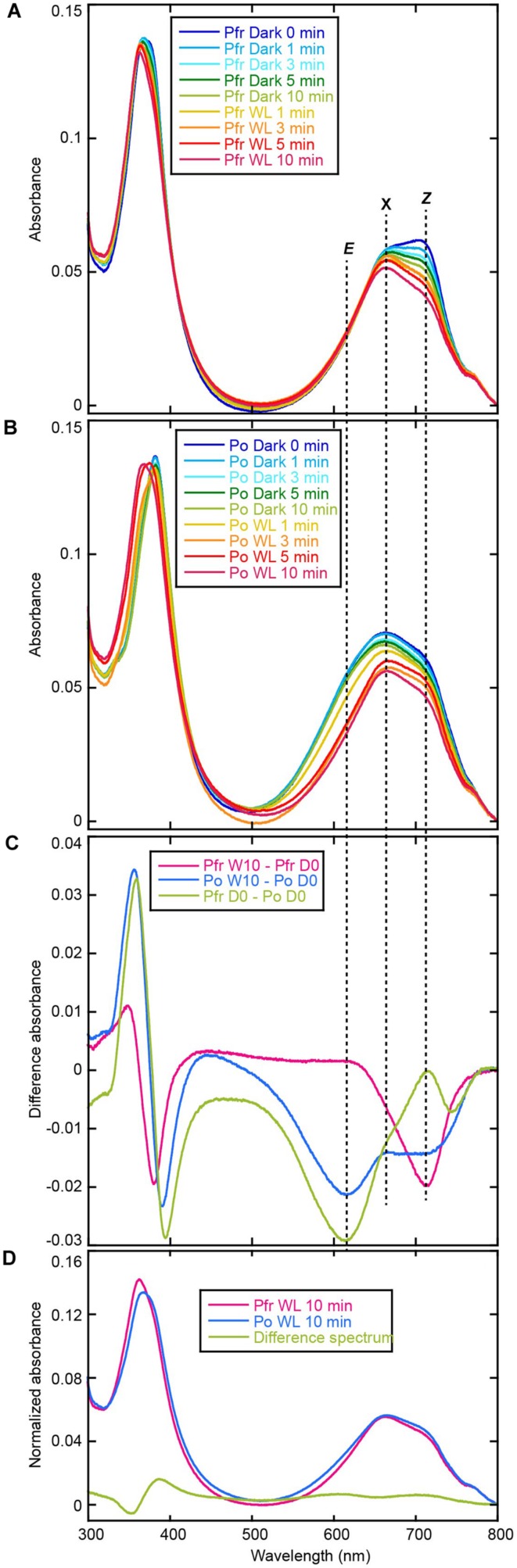
**Acid denaturation of AM1_C0023g2-BV. (A)** Absorption spectra of acid-denatured AM1_C0023g2-BV Pfr during dark and light processes. **(B)** Absorption spectra of acid-denatured AM1_C0023g2-BV Po during dark and light processes. **(C)** Difference spectrum during dark and light processes of denatured Pfr (magenta), during dark and light processes of denatured Po, and of Pfr-minus-Po just after denaturation (green). **(D)** Normalized absorption spectra and difference spectrum of acid-denatured AM1_C0023g2-BV Pfr and Po after dark and light processes.

Acid-denatured spectra of AM1_C0023g2 isolated from BV-producing *E. coli* showed completely different spectra (data not shown) compared with previously reported data (Narikawa et al., 2015b). In order to monitor initial spectral change after denaturation, we denatured samples under 4°C and their absorption spectra were measured at 15°C at 0, 1, 3, 5, and 10 min. Subsequently absorption spectra during white light illumination (1, 3, 5, and 10 min) were recorded. Absorption maximum just after denaturation of Pfr form was observed at ∼700 nm (**Figure 6A**), which corresponded to 15*Z*-BV previously reported (Narikawa et al., 2015b). Absorption maximum after denaturation of Po form was observed at ∼660 nm (**Figure 6B**) with a red-shift compared with 15*E*-BV as reported earlier. Because photoconversion from Pfr to Po is incomplete (**Figure 2B**), the residual Pfr may affect the absorption peak of denatured Po resulting in red shift to ∼660 nm.

The ∼700 nm peak observed for Pfr (denatured) decreased during dark and light processes and final absorption spectrum was at ∼665 nm (**Figure 6A**). Difference spectrum during these processes showed a negative peak at ∼714 nm (**Figure 6C**, Pfr W10 – Pfr D0). From these results, we hypothesized that 15*Z*-BV just after denaturation rapidly converts to an unknown state (X) at 665 nm. On the other hand, absorption after Po denaturation slightly decreased under dark process and largely decreased under light process (**Figure 6B**). The decrease under dark process may correspond to conversion from the residual Pfr to the unknown state “X”. The decrease under light process may correspond to conversion from Po to “X” via Pfr. Based on the above-mentioned hypothesis, in the case of both Pfr denaturation and Po denaturation, their final products after dark and light processes should be composed of only “X”. Indeed, absorption spectra of both denatured Pfr and Po after 10 min white light illumination are at 665 nm and almost same (**Figure 6D**), which strongly supports this hypothesis.

Pfr-minus-Po difference spectrum just after denaturation showed a positive peak around ∼714 nm and a negative peak around ∼614 nm (**Figure 6C**, Pfr D0 – Po D0). These wavelengths well correspond to those obtained from the difference spectrum of denatured AM1_1557g2 (Narikawa et al., 2015b). Thus, the positive peak around 714 nm corresponds to 15*Z*-BV, whereas the negative peak around 614 nm corresponds to 15*E*-BV. However, a negative component around 745 nm was detected. Based on the above-mentioned hypothesis in which the denatured 15*Z*-BV rapidly converts into “X”, 15*E*-BV from denatured Po should be more abundant than 15*Z*-BV from denatured Pfr. In such situation, absorption tail of 15*E*-BV around 745 nm should be larger than that of 15*Z*-BV, and the difference spectrum should show the minus component around 745 nm.

Difference spectrum during light and dark processes of denatured Po showed negative peaks at ∼714 and ∼614 nm (**Figure 6C**, Po W10 – Po D0). These wavelengths also correspond to 15*Z*-BV and 15*E*-BV, respectively. Major negative peak at ∼614 nm is likely to correspond to conversion from 15*E*-BV to “X” via 15*Z*-BV, whereas minor negative peak at ∼714 nm is likely to correspond to conversion from 15*Z*-BV originated in the residual Pfr to “X”.

**FIGURE 7:**
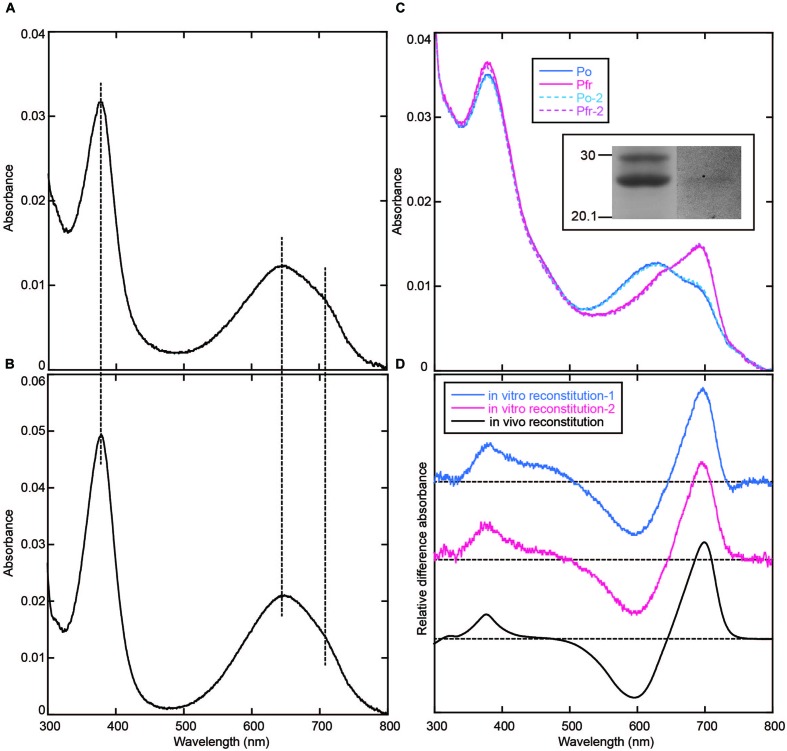
**Denaturation with 1% SDS and *in vitro* reconstitution. (A)** Absorption spectrum of AM1_C0023g2-BV Pfr denatured with 1% SDS. **(B)** Absorption spectrum of AM1_1557g2-BV Pfr denatured with 1% SDS. **(C)** Absorption spectra of the Pfr and Po forms of S334G reconstituted with BV *in vitro*. Photoconversion was repeated twice. First-round Po: Solid blue line, first-round Pfr: Solid magenta line, second-round Po: Dotted cyan line, second-round Pfr: Dotted pink line. Inset: SDS-PAGE of *in vitro* reconstituted S334G. Left, CBB-stained gel. Right, in-gel fluorescence detection. **(D)** Pfr-minus-Po difference spectra of S334G reconstituted *in vitro* and *in vivo*. *In vitro* reconstitution: blue (first-round) and magenta (second-round) lines, *in vivo* reconstitution: black line.

Taking the possibility into consideration that the BV chromophore bound to AM1_C0023g2 is unstable under acidic condition, we denatured the AM1_C0023g2-BV under neutral urea condition. However, the denatured chromophore was also unstable and its absorption was rapidly bleached (data not shown). The result suggests that urea is crucial for the chromophore instability. Thus, we denatured the AM1_C0023g2-BV Pfr under 1% SDS as previously described (Ishizuka et al., 2007). As a result, its absorption maxima was observed at ∼646 nm and almost same as that of AM1_1557g2-BV Pfr (**Figures 7A,B**), indicating that their chromophores are both BV. To further confirm the result, we performed *in vitro* reconstitution analysis using S334G protein and BV chromophore. Although reconstitution efficiency was low, BV covalently bound to S334G was detected by in-gel fluorescence analysis (**Figure 7C**, inset). Far-red/orange reversible photoconversion was observed (**Figure 7C**) and is almost same as that observed for *in vivo* reconstituted one (**Figure 7D**). As a conclusion, the chromophore of AM1_C0023g2 expressed in BV-producing *E. coli* is indeed BV.

### BV Chromophore Instability in the Presence of Urea

Although AM1_C0023g2 binds BV as well as the other BV-binding CBCRs and bacteriophytochromes, BV derived from only AM1_C0023g2 showed unstable nature in the presence of urea. Very recently, Shcherbakova et al. (2015) obtained a fluorescent protein that consists of PAS and GAF domain originally derived from a bacteriophytochrome, RpBphP1 (Shcherbakova et al., 2015). The fluorescent protein, Bph1-FP, covalently binds BV via Cys residue within the GAF domain. The crystal structure revealed that covalent-bonding site of Bph1-FP to BV is heterogeneous, namely C3^1^ or C3^2^. This means that a Cys residue within the GAF domain has a potential to ligate to both C3^1^ and C3^2^. In this context, we hypothesized that the Cys residue of AM1_C0023g2 ligates to C3^1^ and the resultant adduct between the Cys residue and C3^1^ of BV may affect stability of the denatured chromophore in the presence of urea, whereas Cys residues of AM1_1557g2 and AM1_1870g3 are likely to ligate to C3^2^ of BV as well as normal bacteriophytochromes and the resultant chromophore may be stable under the same condition. Just after denaturation of Pfr form, a component corresponding to BV and at 700 nm rapidly converts into a component at 665 nm. This characteristic in absorbing around 665 nm is somehow similar to that of PCB. In this context, BV bound to AM1_C0023g2 may break down into PCB-like chromophore in the presence of urea. Urea may affect conjugated system of BV bound to AM1_C0023g2. In order to verify these hypotheses, we are now trying to crystalize both AM1_1557g2-BV and AM1_C0023g2-BV.

### Evaluation of Chromophore Binding Efficiencies

We calculated binding efficiencies of AM1_C0023g2 to PCB and BV at ∼80 and ∼70%, respectively. Based on these values and in-gel fluorescence analysis (**Figure 1B**), we also estimated the binding efficiencies of AM1_1557g2 to PCB and BV at ∼50 and ∼40%, respectively. The binding efficiencies of AM1_C0023g2 to both PCB and BV are higher than those of AM1_1557g2. The binding efficiency of S334G to BV was calculated to be almost 100%, which is better than that of the wild type protein (∼70%).

Binding efficiency of AM1_C0023g2 to PCB is comparable to or slightly higher than that to BV. In this context, it is intriguing that no BV-binding holoproteins were detected from PCB-producing *E. coli* despite that BV should be present as a precursor. BV may be kept at quite low concentration in the PCB-producing *E. coli* via substrate-channeling from heme oxygenase to PcyA.

### Fluorescence Spectroscopy

**FIGURE 8:**
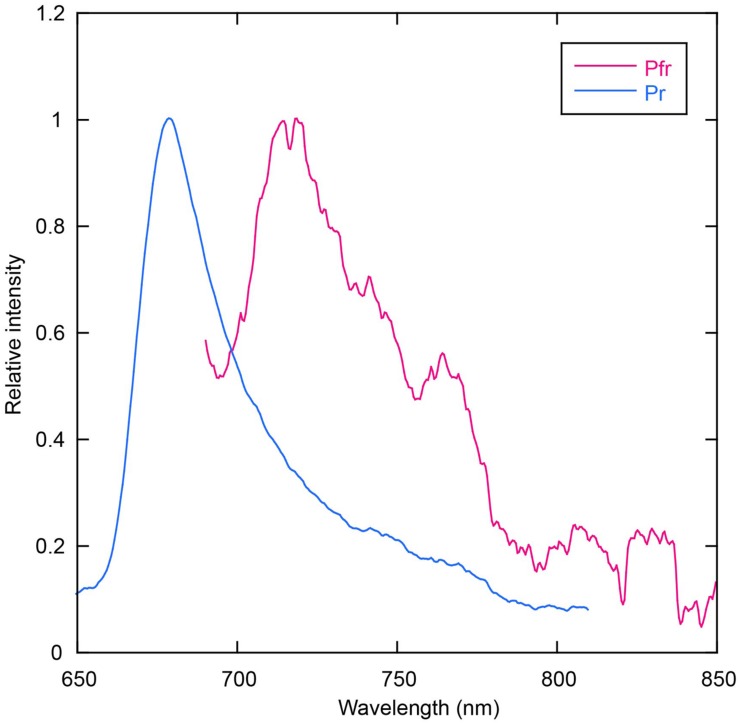
**Fluorescence spectra of AM1_C0023g2-BV Pfr (magenta) and AM1_C0023g2-PCB Pr (blue)**.

Room temperature fluorescence spectra of the thermostable forms of AM1_C0023g2-PCB and AM1_C0023g2-BV were measured to evaluate their potentials as fluorescent imaging probes (**Figure 8**). AM1_C0023g2-BV Pfr form fluoresces with a maximum at 718 nm, whereas AM1_C0023g2-PCB Pr form fluoresces with a maximum at 679 nm. The fluorescence maximum of AM1_C0023g2-BV Pfr is red-shifted by 39 nm compared with that of AM1_C0023g2-PCB Pr, a red shift similar to that found for their absorption spectra maxima. Fluorescence quantum yields of the Pr and Pfr forms were 3.0 and 0.2%, respectively. Fluorescence quantum yield of AM1_C0023g2-PCB Pr is about two times higher than that of AM1_1557g2-PCB Pr (Narikawa et al., 2015b).

### Expression of AM1_1557g2 and AM1_C0023g2 in Mammalian Cells

**FIGURE 9:**
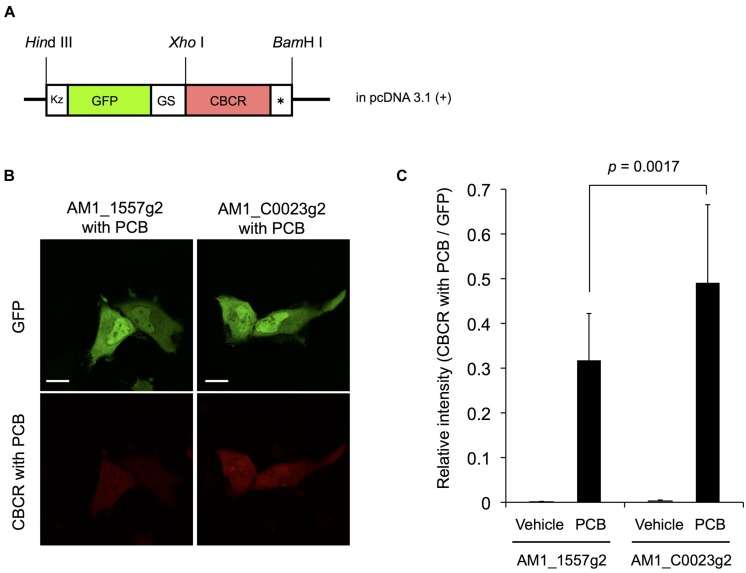
**Expression of GFP-fused AM1_1557g2 and AM1_C0023g2 in mammalian cells. (A)** Schematic diagram of plasmid construct of GFP-fused AM1_1557g2 and AM1_C0023g2. Kz, Kozak sequence for efficient translation in mammalian cells; GS, a flexible peptide linker sequence (Gly-Gly-Ser-Gly-Gly); CBCR, AM1_1557g2 or AM1_C0023g2; ^∗^, stop codon. **(B)** Representative confocal fluorescence images of GFP-fused AM1_1557g2 with PCB and AM1_C0023g2 with PCB. Left column, GFP-fused AM1_1557g2 with PCB; right column, GFP-fused AM1_C0023g2 with PCB; green images, GFP fluorescence; red images, fluorescence of CBCR with PCB. Scale bars, 20 μm. **(C)** Relative intensity of fluorescence of CBCR with PCB normalized to GFP fluorescence. Error bars represent standard deviations (*n* = 16, 16, 16, and 17 for AM1_1557g2 with vehicle, AM1_1557g2 with PCB, AM1_C0023g2 with vehicle and AM1_C0023g2 with PCB, respectively).

We examined the fluorescence of AM1_1557g2-PCB and AM1_C0023g2-PCB in live mammalian cells. To precisely compare the fluorescence intensities of the CBCRs, we fused GFP to the CBCRs as an expression control (**Figure 9A**). HeLa cells were transfected with GFP-fused AM1_1557g2 and AM1_C0023g2 and treated with or without 20 μM PCB. Confocal fluorescence images of GFP-fused AM1_1557g2-PCB and AM1_C0023g2-PCB show bright green fluorescence of GFP, indicating that both GFP-fused AM1_1557g2 and AM1_C0023g2 were successfully expressed in the mammalian cells (**Figure 9B** upper left and right). Almost the same brightness of GFP indicates that the expression levels of GFP-fused AM1_1557g2 and AM1_C0023g2 were similar (**Figure 9B** upper left and right). No near-infrared fluorescence was detected from both cells without addition of PCB (**Figure 9C**). Low fluorescence quantum yield and/or low intrinsic BV level may be the reason for this result. On the other hand, the near-infrared fluorescence was detected from both cells after exposure to PCB. The near-infrared fluorescence of AM1_C0023g2-PCB was much higher than that of AM1_1557g2-PCB (**Figure 9B** lower right and left). Relative intensities of AM1_1557g2-PCB and AM1_C0023g2-PCB normalized to GFP intensities were 0.31 ± 0.10 and 0.49 ± 0.17, respectively (**Figure 9C**). This is the first report detecting fluorescence from PCB-binding CBCRs expressed in the mammalian cells. Their quantum yields are not so high, but comparable to those of the native phytochromes and CBCRs (Sineshchekov, 1995; Chen et al., 2012; Rockwell et al., 2012c; Pennacchietti et al., 2015). Thus, we can consider that AM1_C0023g2-PCB is appropriate for platform of fluorescent probe development as a starting material.

## Author Contributions

RN, MI, and MS designed the research. KF, TN, YA, TY, N-N-W, and RN performed experiments. N-N-W constructed plasmids. KF and RN performed protein purification and spectroscopic analyses. TN and YA performed fluorescence detection from mammalian cells. KF, TN, MS, and RN wrote the manuscript.

## Conflict of Interest Statement

The authors declare that the research was conducted in the absence of any commercial or financial relationships that could be construed as a potential conflict of interest. The reviewer Y-IlP and handling Editor declared a current collaboration and the handling Editor states that the process nevertheless met the standards of a fair and objective review.
